# The Emergence of the New P.4 Lineage of SARS-CoV-2 With Spike L452R Mutation in Brazil

**DOI:** 10.3389/fpubh.2021.745310

**Published:** 2021-10-01

**Authors:** Cíntia Bittar, Fábio Sossai Possebon, Leila Sabrina Ullmann, Dayla Bott Geraldini, Vivaldo G. da Costa, Luiz G. P. de Almeida, Paulo Ricardo da S. Sanches, Nailton M. Nascimento-Júnior, Eduardo M. Cilli, Cecília Artico Banho, Guilherme R. F. Campos, Helena Lage Ferreira, Lívia Sacchetto, Gislaine C. D. da Silva, Maisa C. P. Parra, Marília M. Moraes, Paulo Inácio da Costa, Ana Tereza R. Vasconcelos, Fernando Rosado Spilki, Maurício L. Nogueira, Paula Rahal, João Pessoa Araujo Jr

**Affiliations:** ^1^Laboratório de Estudos Genômicos, Departamento de Biologia, Instituto de Biociências Letras e Ciências Exatas (IBILCE), Universidade Estadual Paulista (Unesp), São José do Rio Preto, Brazil; ^2^Instituto de Biotecnologia, Universidade Estadual Paulista (Unesp), Botucatu, Brazil; ^3^Laboratório de Bioinformática, Laboratório Nacional de Computação Científica (LNCC), Petrópolis, Brazil; ^4^Laboratório de Síntese e Estudos de Biomoléculas (LaSEBio), Departamento de Bioquímica e Química Orgânica, Instituto de Química, Universidade Estadual Paulista (Unesp), Araraquara, Brazil; ^5^Laboratório de Química Medicinal, Síntese Orgânica e Modelagem Molecular (LaQMedSOMM), Departamento de Bioquímica e Química Orgânica, Instituto de Química, Universidade Estadual Paulista (Unesp), Araraquara, Brazil; ^6^Laboratório de Pesquisas em Virologia (LPV), Departamento de Doenças Dermatológicas, Infecciosas e Parasitárias, Faculdade de Medicina de São José do Rio Preto (FAMERP), São José do Rio Preto, Brazil; ^7^Laboratório de Medicina Veterinária Preventiva Aplicada, Departamento de Medicina Veterinária, Faculdade de Zootecnia e Engenharia de Alimentos (FZEA), Universidade de São Paulo (USP), Pirassununga, Brazil; ^8^Departamento de Análises Clínicas, Faculdade de Ciências Farmacêuticas (FCFAR), Universidade Estadual Paulista (Unesp), Araraquara, Brazil; ^9^Laboratório de Microbiologia Molecular, Instituto de Ciências da Saúde, Universidade Feevale, Novo Hamburgo, Brazil

**Keywords:** SARS-CoV-2, COVID-19, lineage P.4, Spike L452R, variants

## Abstract

The emergence of several SARS-CoV-2 lineages presenting adaptive mutations is a matter of concern worldwide due to their potential ability to increase transmission and/or evade the immune response. While performing epidemiological and genomic surveillance of SARS-CoV-2 in samples from Porto Ferreira—São Paulo—Brazil, we identified sequences classified by pangolin as B.1.1.28 harboring Spike L452R mutation, in the RBD region. Phylogenetic analysis revealed that these sequences grouped into a monophyletic branch, with others from Brazil, mainly from the state of São Paulo. The sequences had a set of 15 clade defining amino acid mutations, of which six were in the Spike protein. A new lineage was proposed to Pango and it was accepted and designated P.4. In samples from the city of Porto Ferreira, P.4 lineage has been increasing in frequency since it was first detected in March 2021, corresponding to 34.7% of the samples sequenced in June, the second in prevalence after P.1. Also, it is circulating in 30 cities from the state of São Paulo, and it was also detected in one sample from the state of Sergipe and two from the state of Rio de Janeiro. Further studies are needed to understand whether P.4 should be considered a new threat.

## Introduction

The emergence of SARS-CoV-2 at the end of 2019 has led to over 4 million deaths worldwide so far (1). Coronavirus is a group of enveloped, single-stranded positive RNA viruses which infects mammals and birds (2). Molecular data suggest that SARS-CoV-2 originated in bats, as has the other *Betacoronavirus* that cause severe respiratory syndrome SARS-CoV and MERS-CoV, and emerged in humans following passage through an intermediary host (3, 4).

Viruses from the *Coronaviridae* family have unusually large genomes for RNA viruses (26 to 30 kb) (5). This is only possible due to the exonuclease activity of Nsp14 protein responsible for proofreading the RNA during replication, which maintains genome stability and a mutation rate of ~10^−6^ mutations/site/cycle (6–8). Despite the low mutation rate, the intense circulation of the virus favors the appearance of mutations in the genomes leading to the development of many different lineages (9, 10).

The emergence of lineages bearing adaptive mutations has raised concerns worldwide since they increase viral fitness and have rapidly spread and replaced previously circulating viruses (11–13). B.1.1.7 (Alpha), B.1.351 (Beta), Lineages P.1 (Gamma), and B.1.617.2 (Delta, a L452R mutant) are the current Variants of Concern (VOC) (14). They all present important mutations in Spike protein, especially in the Receptor Binding Domain (RBD) region, that have shown to reduce recognition by neutralizing antibodies *in vitro* (15). Although, studies show that the vaccines available so far are still effective against these new variants, as the virus continues to circulate at high rates, new adaptive mutations can emerge compromising the immunization of the population (16, 17). Thus, genomic surveillance is essential to identify new mutations in the existing VOCs as well as new emerging lineages that might pose a threat.

While doing epidemiological and genomic surveillance of SARS-CoV-2 in samples from the city of Porto Ferreira—SP—Brazil, using Sanger and NGS techniques, we detected a new lineage designated by Pango as P.4, harboring the L452R mutation, that is circulating in São Paulo state (18). This new variant may be regarded as a VOI and we should maintain a close look in its dissemination and evolution.

## Materials and Methods

### Samples

This study is a part of the Corona-ômica.BR/MCTI Network and included RT-qPCR positive nasopharyngeal swab (NPS) samples from subjects from the city of Porto Ferreira—SP—Brazil, that were sent to the Instituto de Biotecnologia, UNESP, Botucatu for SARS-CoV-2 diagnosis. Samples collected from January to June with Cq levels lower than 27 were selected for genomic surveillance (19). This study was approved by the Institutional Ethical Review Board (protocol number: 33202820.7.1001.5348), following Brazilian regulations and international ethical standards.

### RNA Extraction

Purified RNA was obtained with Guanidine Isothiocyanate cell lysis and nuclease inactivation, followed by magnetic beads purification (20) After diagnosis, aliquots of purified RNA samples were kept in −80°C storage until further analysis.

### Tracking Variants by Sanger Sequencing

A Sanger sequencing strategy was designed to differentiate the main variants of concern (VOC) that have been circulating in Brazil. A set of PCR primers (SARS-CoV-2_S1_PF 5′ GAGTCCAACCAACAGAATC 3' and SARS-CoV-2_S1_PR 5′ GAATCTCAAGTGTCTGTGG 3′) was designed to amplify a fragment of the Spike genomic region (nt 956 to nt 1753), comprising the Receptor Binding Domain (RBD). cDNA was synthesized using the High-Capacity cDNA Reverse Transcription Kit (Applied Biosystems) replacing random primers by the specific SARS-CoV-2_S1_PR primer. The PCR reaction was performed using GoTaq G2 Green Master Mix (Promega) and the specific primers. The sequencing reactions were set up in duplicates using SARS-CoV-2_S1_PF primer and BigDye Terminator v3.1 Cycle Sequencing Kit (Applied Biosystems) and readings were made in ABI 3130xl Genetic Analyzer (Applied Biosystems).

### Sanger Sequences Analyses

Partial Spike sequences obtained by Sanger sequencing were submitted to Electropherogram Quality Analysis for quality check and contig assembly (21). Sequences were aligned with the GISAID reference sequence EPI_ISL_402124 (only Spike) using Clustal Omega available at Seaview 4.6.1 (22, 23). The alignment was analyzed using BioEdit 7.2.5 (24). By analyzing four codons it is possible to differentiate between B.1.1.7, P.1, B.1.351 and P.2/N.9 as can be seen in Supplementary Figure 1. The analysis of the fragment also allows detecting new mutations in the RBD region.

### Next Generation Sequencing (NGS)

NGS was used to confirm mutations found in Sanger screening. Libraries were prepared with the COVIDSeq^TM^ Illumina Test (Illumina Inc., San Diego, CA, USA), according to the manufacturer's instructions. Briefly, 8.5 μL of RNA was used as input for cDNA synthesis, followed by amplification with two sets of primers covering the entire SARS-CoV-2 genome, tagmentation, and amplification. Libraries were then pooled and purified. The final pool was quantified with the Qubit DNA High Sensitivity kit (Thermo Fisher Scientific, Waltham, MA, USA), denatured and sequenced in 600 cycles V3 flow cells (2 x 151 cycles) with the MiSeq System (Illumina Inc.) at 10 pM and 10% PhiX.

### NGS Reads Analysis and Lineage Classification

The Illumina^TM^ DRAGEN COVID Lineage workflow (Illumina Inc., San Diego, CA, USA—Available at https://www.illumina.com/products/by-type/informatics-products/dragen-bio-it-platform) was used to assemble NGS reads and generate consensus sequence. Sequences were classified using Pango lineages assignment tool (pangolin) (18).

### Mutation Analysis

Preliminary mutation analyses were performed using Nextclade (25). To determine the set of characterizing mutations present in the potential new lineage, a database containing all mutations present in 2,770 genomes classified as B.1.1.28 was constructed (downloaded from GISAID June 4, 2021—acknowledgments in Supplementary File 1). The variant calling and annotation were performed with the snpsites v2.3.3 and SnpEff/snpSift 4.5covid19 programs, respectively. Using the database, we searched for all genomes that contain the T22917G (L452R) mutation. Next, in the set of genomes found, we verified all mutations, excluding those that define the B.1.1.28 lineage (C241T, F924F, P4715L, D614G, V1176F, R203K, R203R and G204R).

### Phylogenetic Analysis

Two datasets were assembled for phylogenetic analysis. The first is composed of complete genome sequences generated in this study along with high coverage sequences available in GISAID of B.1.1.28 lineage with L452R mutation as well as representatives from B.1.1.28, P.1, P.2, P.3, B.1.1.7, B.1.427, B.1.429, B.1.617 lineages plus the reference sequence WIV04/2019|EPI_ISL_402124. The second is composed of all high coverage sequences, assigned by Pango as P.4, available in GISAID up until August 28, 2021. The datasets were aligned in MAFFT 7, available at https://mafft.cbrc.jp/alignment/server/, using default parameters. Both ends of the alignments were trimmed based on the smaller sequence using BioEdit. Maximum likelihood phylogenetic analyses were performed using PhyML 3.0 hosted at ATGC Montpellier Bioinformatics Platform (26). The substitution model was estimated by Smart Model Selection (SMS) implemented in PhyML and branch support was calculated by aLRT (27, 28). The phylogenetic tree based on the first dataset was edited using FigTree 1.1.4 (29). The P.4 phylogeny, based on the second dataset, was associated with geographical and temporal data using Microreact (30).

### Modeling the Structure of SARS-CoV-2 Spike Protein From P.4 and Its Interaction With ACE2 Receptor

The structure of the Spike protein from P.4 lineage was modeled using SWISS-MODEL (31). A FASTA amino acid sequence of the Spike protein was loaded and a structure of the Spike protein from SARS-CoV-2 (Wuhan) bounded to the angiotensin-converting enzyme 2 (ACE2) was identified as the most appropriated template (PDB ID: 7DF4) for the computational study, presenting resolution of 3.80 Å2 (32). The 7df4.pdb file was downloaded from Protein Data Bank website and was used as template for modeling (33). The interaction between the P.4 Spike protein was analyzed by PyMol Molecular Graphics System (version: 2.3.2) and compared with the Spike from Wuhan strain (34).

## Results

Porto Ferreira is a city with 56,504 habitants and reported 6,720 Covid-19 cases and 149 deaths by COVID-19 as of the date this manuscript was written. From a total of 2,515 exams of RT-qPCR in NPS samples received by Instituto de Biotecnologia from Porto Ferreira city for Sars-CoV-2 detection, between February and June of 2021, 439 were positive and eligible for variant tracking by Sangersequencing. We detected the circulation of lineages P.1 (*n* = 302), B.1.1.7 (*n* = 18) and P.2/N.9 (*n* = 23). Also, 96 sequences presented only the mutation L452R in the analyzed fragment.

The L452R mutation had not been reported in any endogenous lineage circulating in Brazil at the time of the study (variant Delta was only introduced in the country later), so it required further investigation, through complete genome analysis, to understand which lineage we were detecting. Following, we sequenced the complete genome of 251 samples from Porto Ferreira from those that were previously screened by Sanger, being 92 samples that presented the L452R mutation, initially classified by pangolin as B.1.1.28, 142 classified as Gamma (P.1), and 14 classified as Alpha (B.1.1.7).

**Figure 1:**
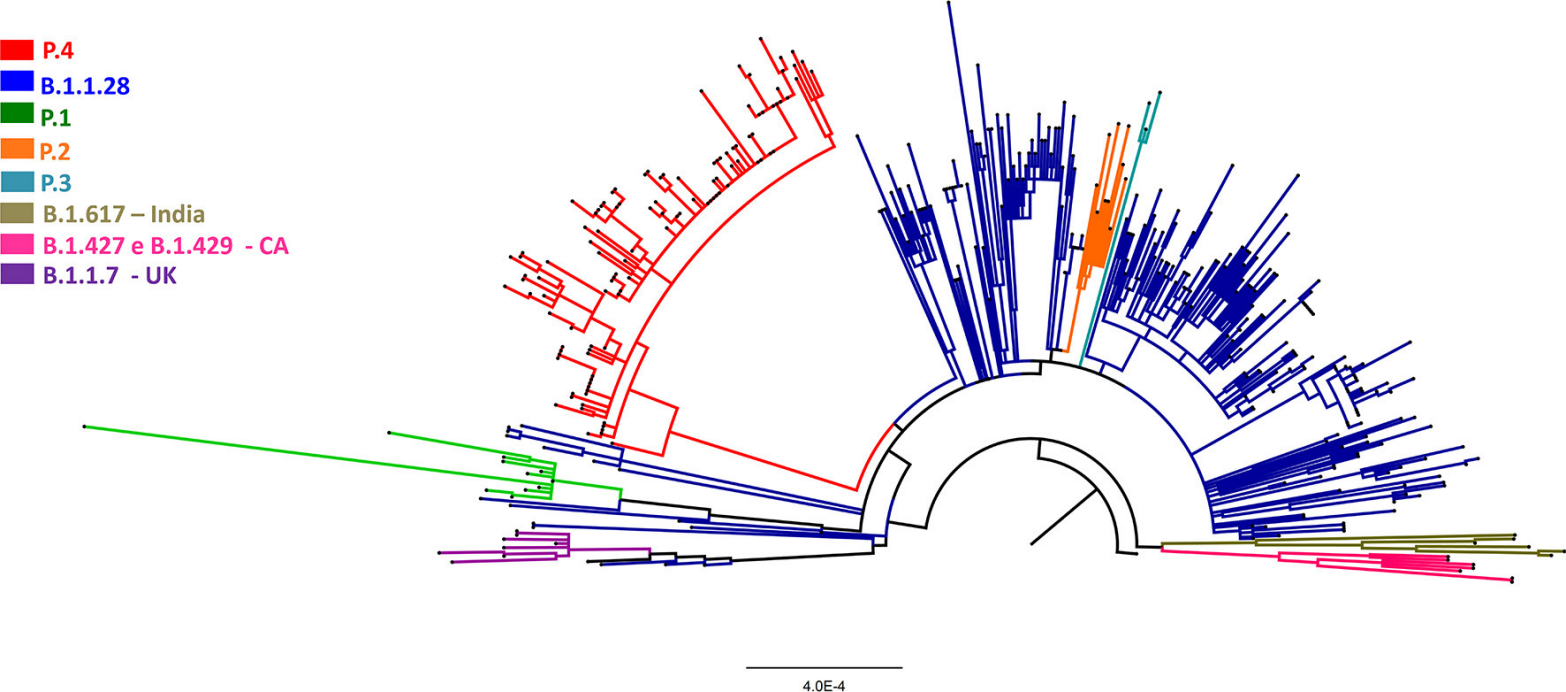
Maximum Likelihood Phylogenetic tree based on a dataset of 407 complete genome sequences of SARS-CoV-2. Tree was reconstructed based on GTR+G+I model and branch support calculated by aLRT (See Supplementary Figure 2 for detail on P.4 branch).

We performed a phylogenetic analysis using the Maximum Likelihood method, using the *GTR*+*G*+*I model*, with only high coverage sequences based on a dataset of 407 sequences. The dataset included sequences harboring the L452R mutation from this study together with representatives of B.1.1.28, P.1, P.2, P.3, B.1.1.7, B.1.427, B.1.429, B.1.617 lineages and the reference sequence WIV04/2019|EPI_ISL_402124 available on GISAID (see Supplementary File 2 for sequence information). The analysis revealed that sequences from Porto Ferreira, with L452R mutation, grouped into a monophyletic branch, with strong branch support (aLRT 1) along with other Brazilian sequences deposited on GISAID carrying the same mutation (Figure 1; Supplementary Figure 2). Most sequences of this branch belonged to other 28 cities from São Paulo state.

We also analyzed the set of mutations shared by the sequences that composed the clade, to verify the defining mutations of this possible new variant. Results showed that most of the sequences share 21 nucleotide mutations, resulting in 15 amino acid substitutions that are not present together in other B.1.1.28 sequences (Table 1). Six of those mutations occur in the Spike protein, but only L452R is in the RBD region. We submitted a proposal to Pango to consider this clade as a new lineage. The proposal was accepted and the new lineage was designated as P.4 (35).

**Table 1:** Lineage P.4 defining mutations.

**Genomic region**	**Nucleotide**	**Amino acid**
	A136G	–
ORF1a	G1811A	A516T
ORF1a	C3177T	P971L
ORF1a	C9693T	A3143V
ORF1a	T9867C	L3201P
ORF1a	C11450A	Q3729K
ORF1a	C12008T	L3915F
ORF1a	C12880T	–
ORF1b	A15932G	Y822C
ORF1b	G18756T	–
ORF1b	C20436T	–
S	G21987T	G142V
S	T22054G	N164K
S	C22079A	Q173K
S	T22917G	L452R
S	C23673T	S704L
S	A23720G	I720V
ORF3a	G25540A	V50I
ORF8	C28253T	–
	A28271C	–
N	C28932T	A220V

**Figure 2:**
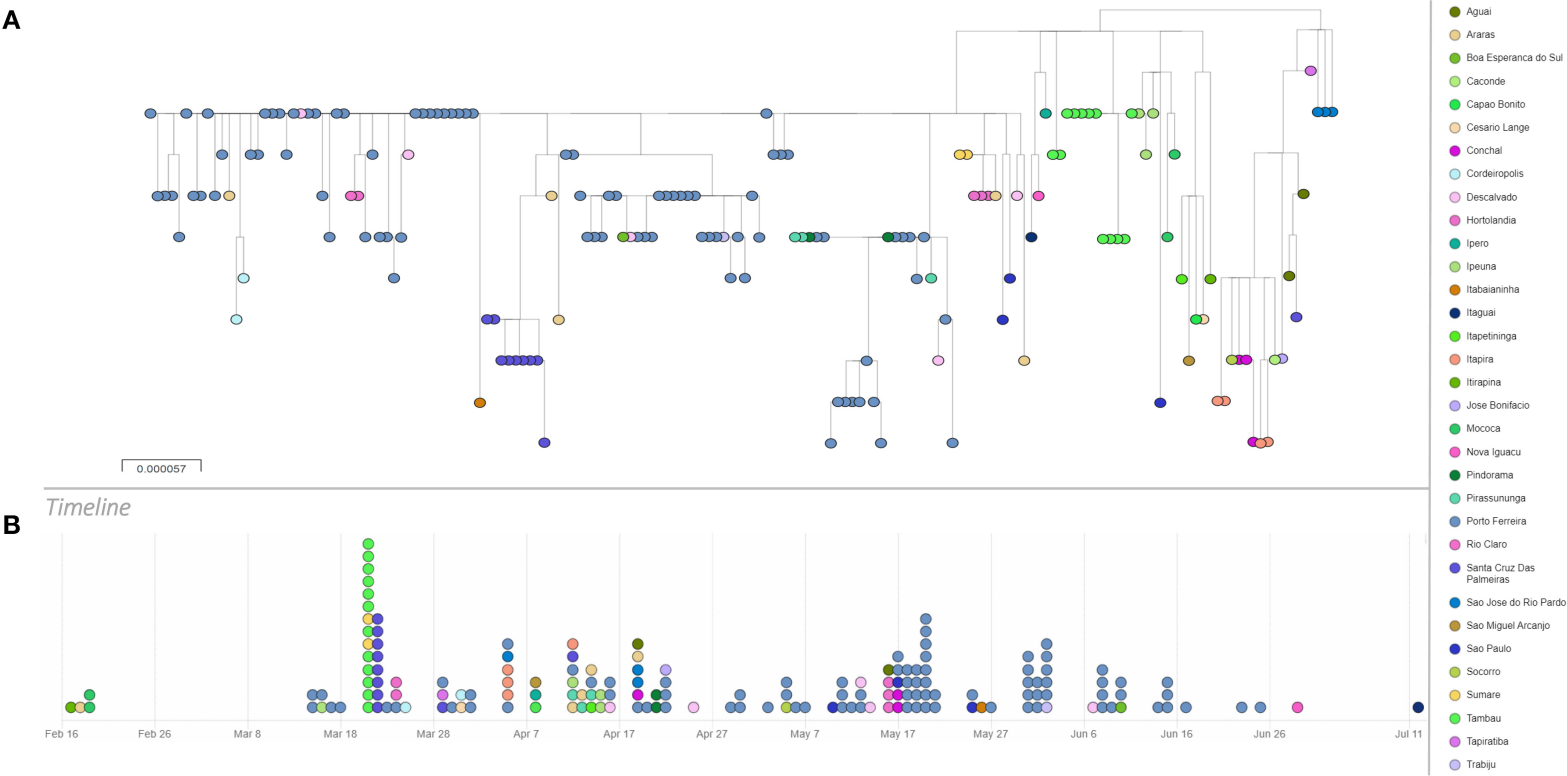
Phylogeny and timeline of P.4 lineage. **(A)**. Maximum Likelihood Phylogenetic tree based on a dataset of 166 complete genome P.4 lineage sequences of SARS-CoV-2. Tree was reconstructed based on GTR+G+I model. **(B)**. Timeline of the detection of lineage P.4. This analysis is available at https://microreact.org/project/3K5w6sxdF2SnDNADdnwDmC/981f0278.

**Figure 3:**
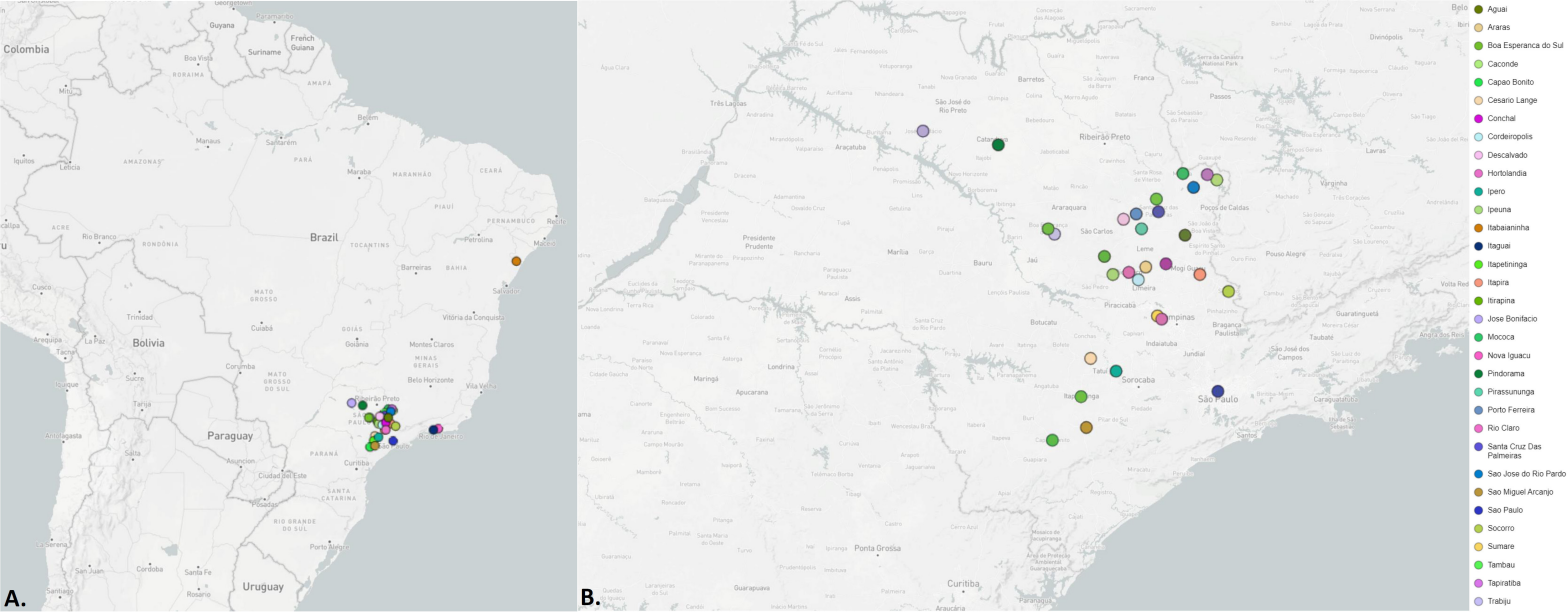
Geographical location of detection of P.4 lineage. **(A)**. Brazil. **(B)**. State of São Paulo.

Following, we did a phylogenetic analysis based on a dataset that includes all high coverage sequences, classified by Pango as P.4, available on GISAID until late August 2021 and associated with geographical and temporal data using Microreact (See Supplementary File 3 for sequence information) (30). Results show that although P.4 lineage has been mostly reported in the state of São Paulo it was also detected in other two Brazilian states, Sergipe (May) and Rio de Janeiro (June and July) (Figures 2, 3).

**Figure 4:**
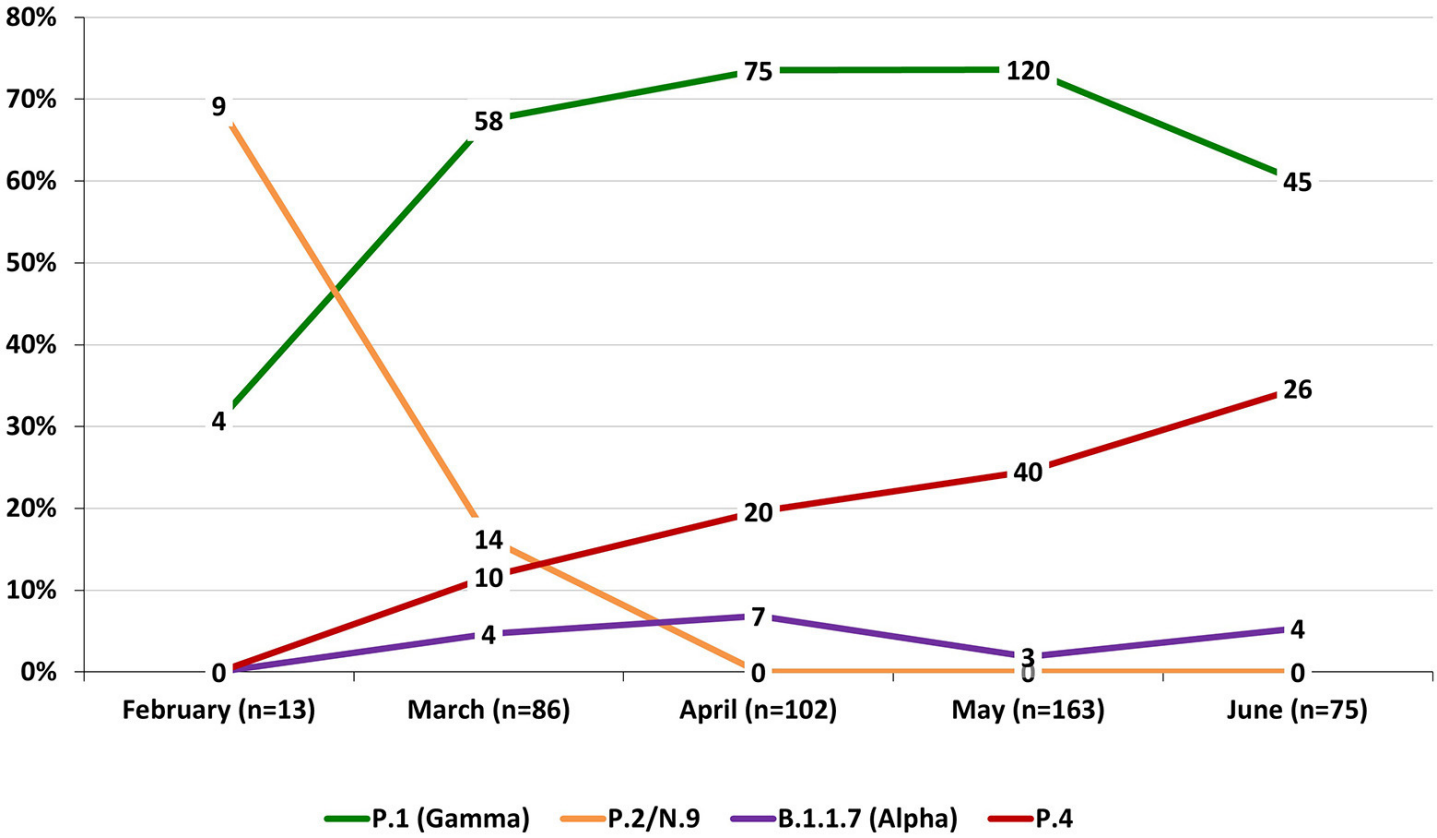
Distribution of lineages throughout the months in the city of Porto Ferreira—SP. Data from nasopharyngeal swab samples received by Instituto de Biotecnologia—Universidade Estadual Paulista (UNESP) for SARS-CoV-2 surveillance.

Considering that most sequences from lineage P.4 are from Porto Ferreira—SP, we analyzed the distribution of all the lineages in this city. The P.4 lineage increased in frequency after its emergence in March when 67.4 and 16.3% of detected lineages were assigned as P.1 and P.2, respectively. The P.4 prevalence increased in the following months, reaching 34.7 % (26/75) in June, representing the second most prevalent after P.1 (Figure 4).

**Figure 5:**
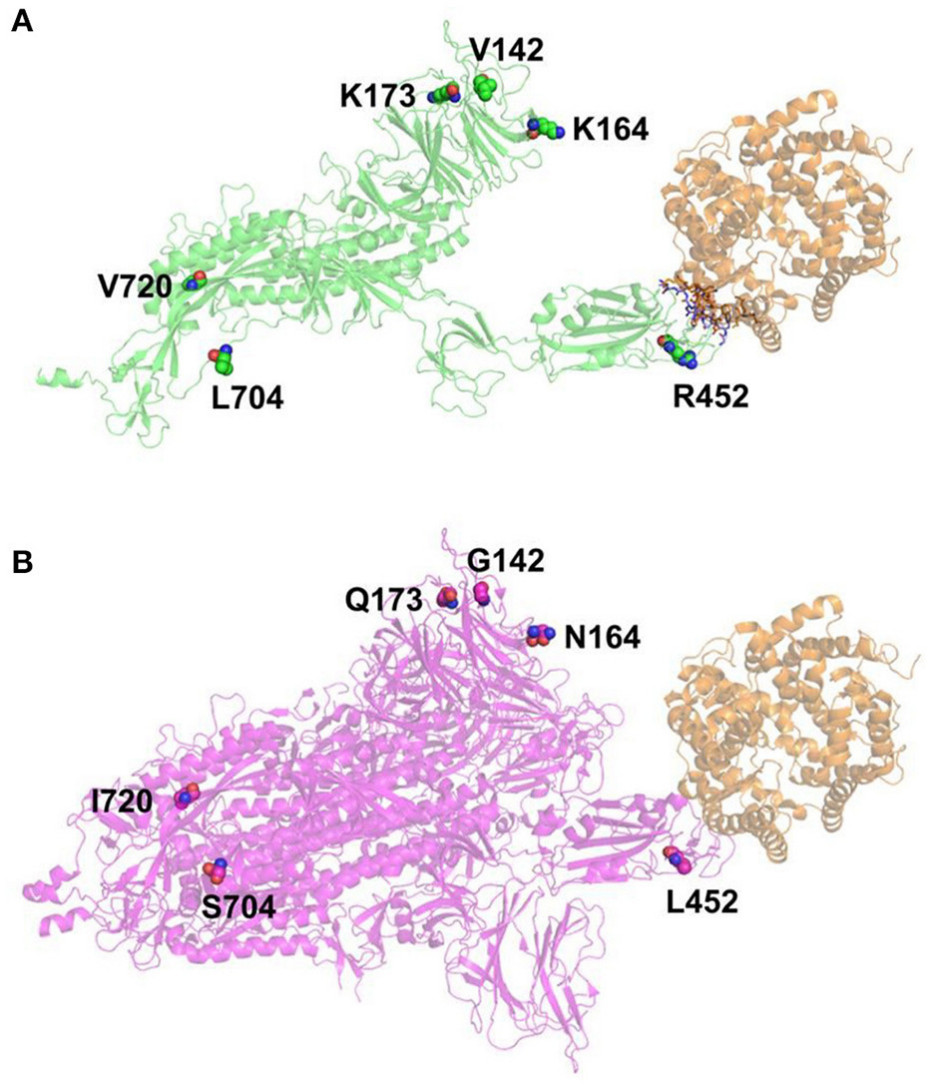
Positions of the mutated residues (presented in spheres, with nitrogen atoms in blue and oxygen atoms in red) in the SARS-CoV-2 Spike proteins. **(A)**. SARS-CoV-2 P.4 lineage (ribbon in green). **(B)**. SARS-CoV-2 Wuhan ribbon in magenta as a trimer. ACE2 is presented in orange (ribbon) for both complexes. The figure was generated using PyMol software.

**Figure 6:**
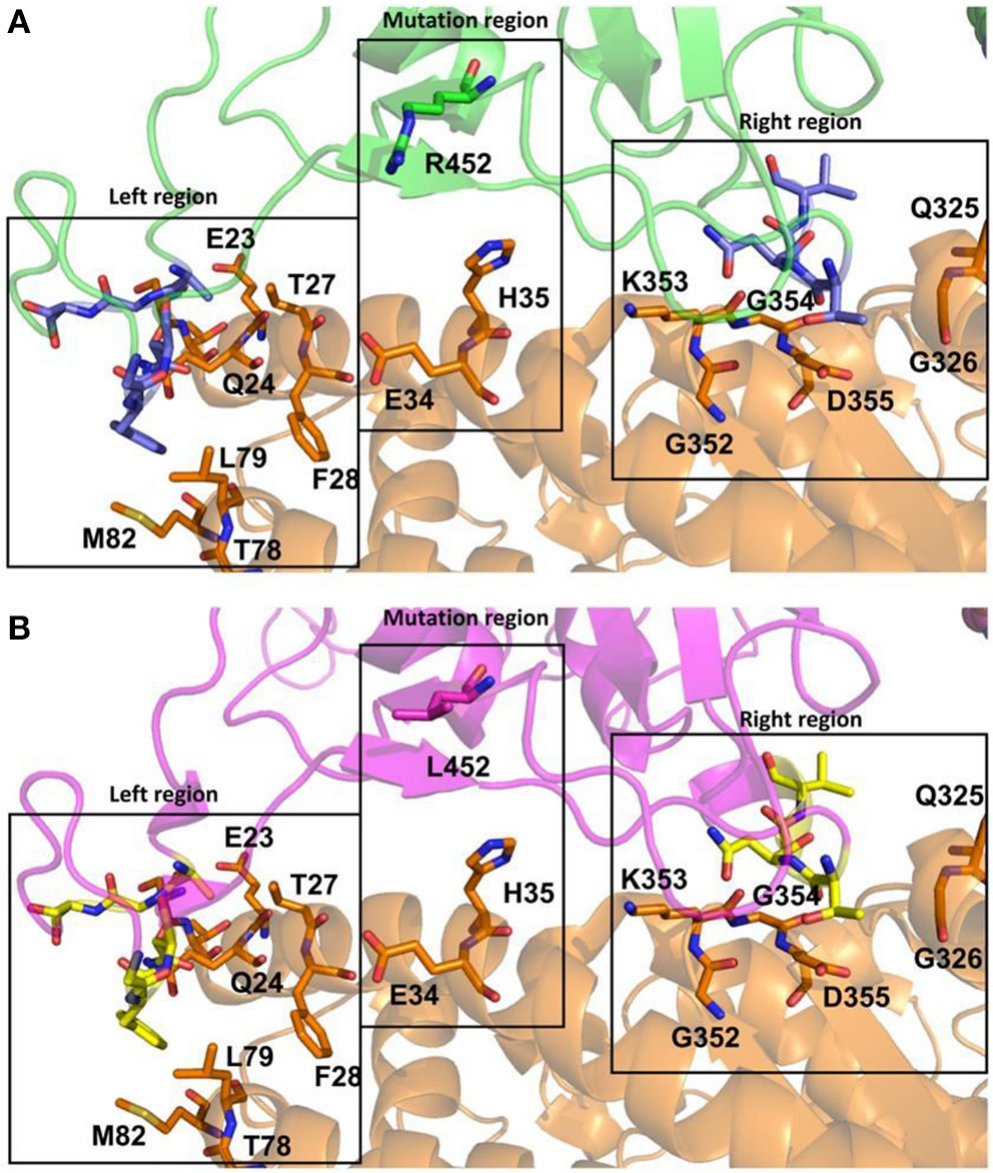
The interaction regions between the SARS-CoV-2 Spike proteins and the ACE2 receptor (ribbons and sticks in orange). **(A)**. SARS-CoV-2 Spike protein for the P.4 lineage (*in silico* model) is highlighted in green (ribbons and sticks, including the mutated residue), with important conserved residues in slate (sticks). **(B)**. SARS-CoV-2 Spike protein for the Wuhan glycoprotein (cryo-EM structure) is highlighted in magenta (ribbon and sticks, including the mutated residue), with important conserved residues in yellow (sticks). For all atoms presented in sticks, carbon atoms are shown in orange (ACE2) or green/slate/magenta/yellow (Spike proteins), nitrogen atoms are shown in blue, oxygen atoms are shown in red and all hydrogen atoms are omitted for clarity. The figure was generated using PyMol software.

The interaction between the Spike protein of P.4 lineage with the ACE2 receptor was modeled and compared with the Wuhan original strain. Figure 5 illustrates the position of the mutation present on the Spike protein: G142V, N164K, Q173K, L452R, S704L, and I720V. The region of interaction between the Spike protein and ACE2 receptor is presented in Figure 6. We have arbitrarily divided this region in three parts in order to aid the analysis: (i) the “Left region” comprising E23, Q24, T27, F28, T78, L79, and M82 residues of ACE2 (in orange), (ii) the “Right region” comprising Q325, G326, G352, K353, G354 and D355 residues of ACE2 and (iii) the “Mutation region” comprising E34 and H35 residues of ACE2. Both SARS-CoV-2 Spike proteins present the same residues in the “Left region” and in the “Right region,” where carbons are presented in late for P.4 lineage (Figure 6A) and in yellow for Wuhan (Figure 6B) Spike proteins. These residues are in the regions which are responsible for Spike-ACE2 interactions for both SARS-CoV-2 Spike proteins. The “Mutation Region” highlights the R452 residue in the P.4 Spike protein (in green, Figure 6A) which may be able to perform electrostatic and cation-π interactions with E34 and H35 (respectively) depending on the conformational Spike-ACE2 changes. These strong potential interactions are not possible for the L452 residue in the Wuhan Spike protein (in magenta, Figure 6B).

## Discussion

The explosive circulation of SARS-CoV-2 since its emergence in late 2019 has allowed the virus to diversify, giving rise to hundreds of different lineages. Some of those present adaptive mutations with considerable impact in transmission efficiency and potential reduction on the effectiveness of vaccines (15).

Through genomic surveillance of lineages circulating in the state of São Paulo—Brazil, we detected the circulation of a new lineage of SARS-CoV-2 in the city of Porto Ferreira bearing the L452R mutation as well as other 14 non-synonymous mutations, not found together in other SARS-CoV-2 sequences. Further analyses, based on our sequences and other sequences deposited on GISAID, revealed that the new variant is circulating in different cities from the state of São Paulo and was also detected in the states of Sergipe and Rio de Janeiro. This lineage descends from B.1.1.28, as has P.1 and P.2 (Zeta) lineages, both of which also emerged in Brazil and was named P.4.

Although we do not know at this time where or how P.4 lineage emerged, persistent circulation is known to be a major driver of emergence of adaptive mutations, which in turn favors the appearance of new lineages. In this case, lineages emerge by a multi-step process, where mutations rise one at a time, along several infections. An alternative hypothesis would be that long-term infections, especially in immunocompromised individuals, could provide a favorable environment for the rapid accumulation of several mutations, being a potential source of emergence of new lineages (36). Studies on lineages Gamma (P.1) and Beta (B.1.351) indicate that they emerged through sequential steps (37, 38). Considering the increase in the number of cases of COVID-19 in early 2021 in Brazil with social distancing levels lower than 40% in the state of São Paulo (1, 39), and limited NGS data, it is possible that intermediate sequences, harboring a subset, of mutations were not sequenced. It is therefore likely that the emergence of P.4 is a result of increased viral circulation due to low social distancing and low vaccination rates.

We analyzed how the mutations found in P.4 could interfere in Spike-ACE2 recognition. Results showed that the mutation of a Leucine (L) to an Arginine (R) in residue 452 has an impact since it allows additional interactions between Spike protein and the cell receptor. The other five mutations present in Spike protein from P.4 lineage did not affect the interaction with this receptor, which was expected since they are not located in the RBD region. The mutation L452R has been shown to decrease sensitivity to neutralizing antibodies in previous studies, when compared with parental strains, and has been emerging independently by convergent evolution in different lineages like the VOCs B.1.427/B.1.429 (Epsilon) and B.1.617.2 (Delta) (40–43). This mutation has been increasing in frequency worldwide since November 2020, being the second most frequent mutation up until July 2021, preceded only by N501Y (44).

Although we do not know at this time how P.4 will behave and if it will pose a significant problem, it certainly raises some concerns. Our analysis in Porto Ferreira—SP showed that P.4 emerged in a scenario of predominance of P.1 and has increased in frequency corresponding to 34.7 % of the samples analyzed in June 2021. P.1 is a highly aggressive lineage that has been shown to replace previously circulating lineages, in a short time, following its introduction in cities from Brazil (37, 45). In fact, it is the most prevalent lineage in all regions of the state of São Paulo (46). Our results revealed that P.4 is circulating in at least 30 cities of the state, suggesting an ability to compete with P.1. Unfortunately, the lack of epidemiological data prevented us to correlate the emergence of P.4 and the number of cases, severity and death in the city.

In summary, we report the emergence of a new lineage called P.4 of SARS-CoV-2 derived from B.1.1.28. Further studies are necessary to understand how transmissible and pathogenic P.4 lineage is and if or how it will impact the immunization of the population and/or pathogenicity of the disease.

## Data Availability Statement

The datasets presented in this study can be found in online repositories. The names of the repository/repositories and accession number(s) can be found in the article/Supplementary Material.

## Ethics Statement

The studies involving human participants were reviewed and approved by the Institutional Ethical Review Board (protocol number: 33202820.7.1001.5348), following Brazilian Regulations and International Ethical Standards. Written informed consent from the participants' legal guardian/next of kin was not required to participate in this study in accordance with the national legislation and the institutional requirements.

## Author Contributions

CB, FSP, MLN, PR, HLF, and JPAJ: conceptualization. CB, FSP, and LGPA: methodology. CB, FSP, LGPA, PRSS, NMNJ, and EMC: formal analysis. CB, FSP, LSU, DBG, VGC, LGPA, CAB, GRFC, LS, GCDS, MCPP, and MM: investigation. MLN, PR, HF, PC, and JA: resources. CB: writing—original draft preparation. FSP, LSU, ATRV, MLN, FRS, PR, HLF, and JPAJ: writing—review and editing. MLN, PR, HLF, PIC, and JPAJ: supervision. MLN, PR, HLF, PIC, and JPAJ: project administration. AV, FS, ATRV, FRS, and JPAJ: funding acquisition. All authors contributed to the article and approved the submitted version.

## Funding

This work was developed in the framework of Rede Corona-ômica BR MCTI/FINEP affiliated to RedeVírus/MCTI (FINEP 01.20.0029.000462/20, CNPq 404096/2020-4). NMNJ is supported by FAPESP (2018/00187-7). EMC is supported by FAPESP (2020/12519-4; 2020/05761-3). GRFC is supported by FAPESP (2020/07419-0). MLN is supported by a FAPESP COVID Grant (20/04836-0). MLN is partially supported by a NIH Grant (CREATE-NEO 1 U01 AI151807-01). MLN is a CNPq Research Fellow. COVID research in MLN's lab is supported by a kindly donation from JBS. ATRV is supported by CNPq (303170/2017-4) and FAPERJ (E-26/202.903/20).

## Conflict of Interest

The authors declare that the research was conducted in the absence of any commercial or financial relationships that could be construed as a potential conflict of interest.

## Publisher's Note

All claims expressed in this article are solely those of the authors and do not necessarily represent those of their affiliated organizations, or those of the publisher, the editors and the reviewers. Any product that may be evaluated in this article, or claim that may be made by its manufacturer, is not guaranteed or endorsed by the publisher.
