# Supplementary figures and images for: The Emergence of the New P.4 Lineage of SARS-CoV-2 With Spike L452R Mutation in Brazil
Source: Front Public Health. 2021 Oct 1;9:745310. doi: 10.3389/fpubh.2021.745310 (PMC8517261; doi:10.3389/fpubh.2021.745310)

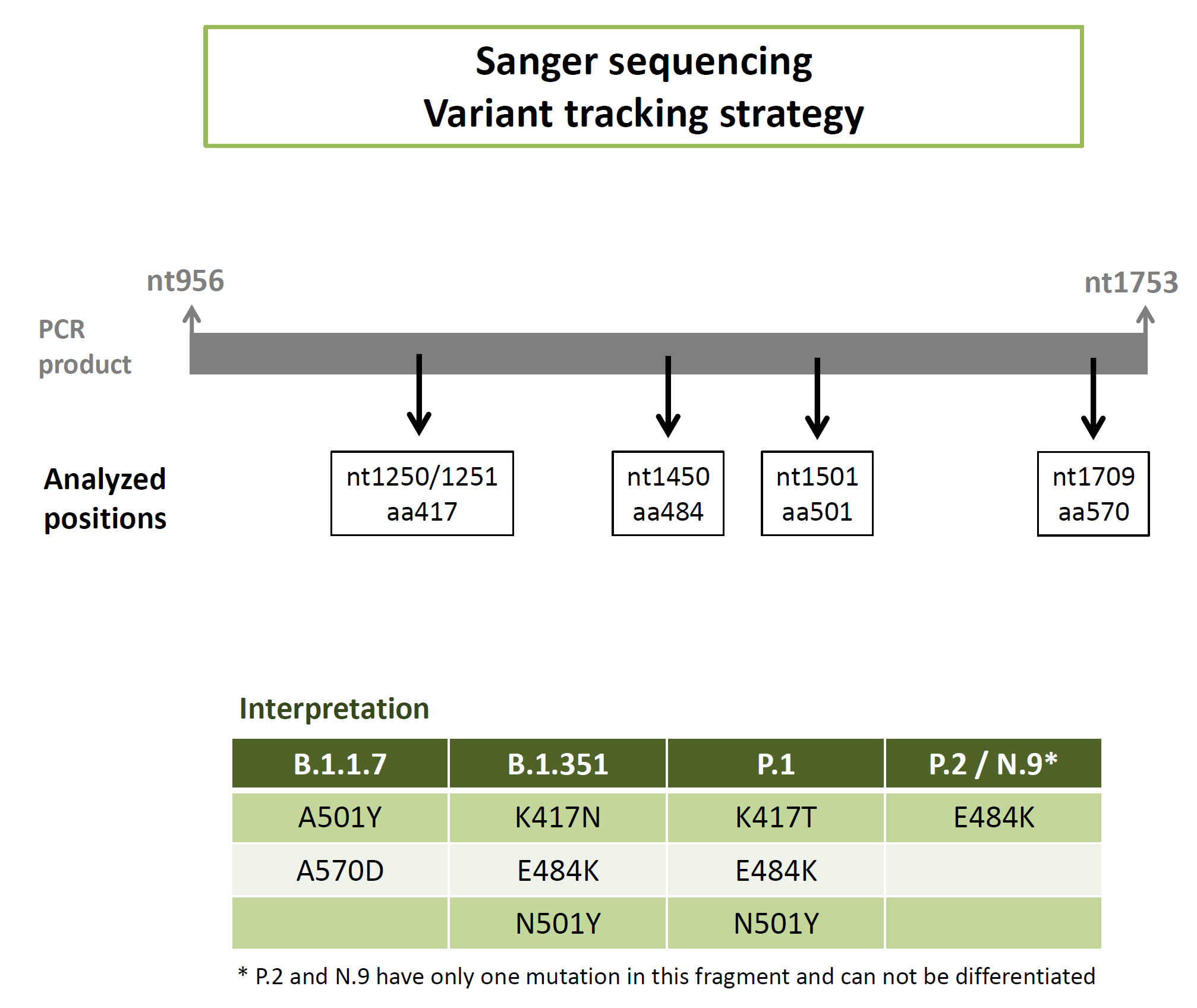

Supplement: Supplementary file 1 [file Data_Sheet_1.zip › Supplentary/Supplementary Figure 1.tif]

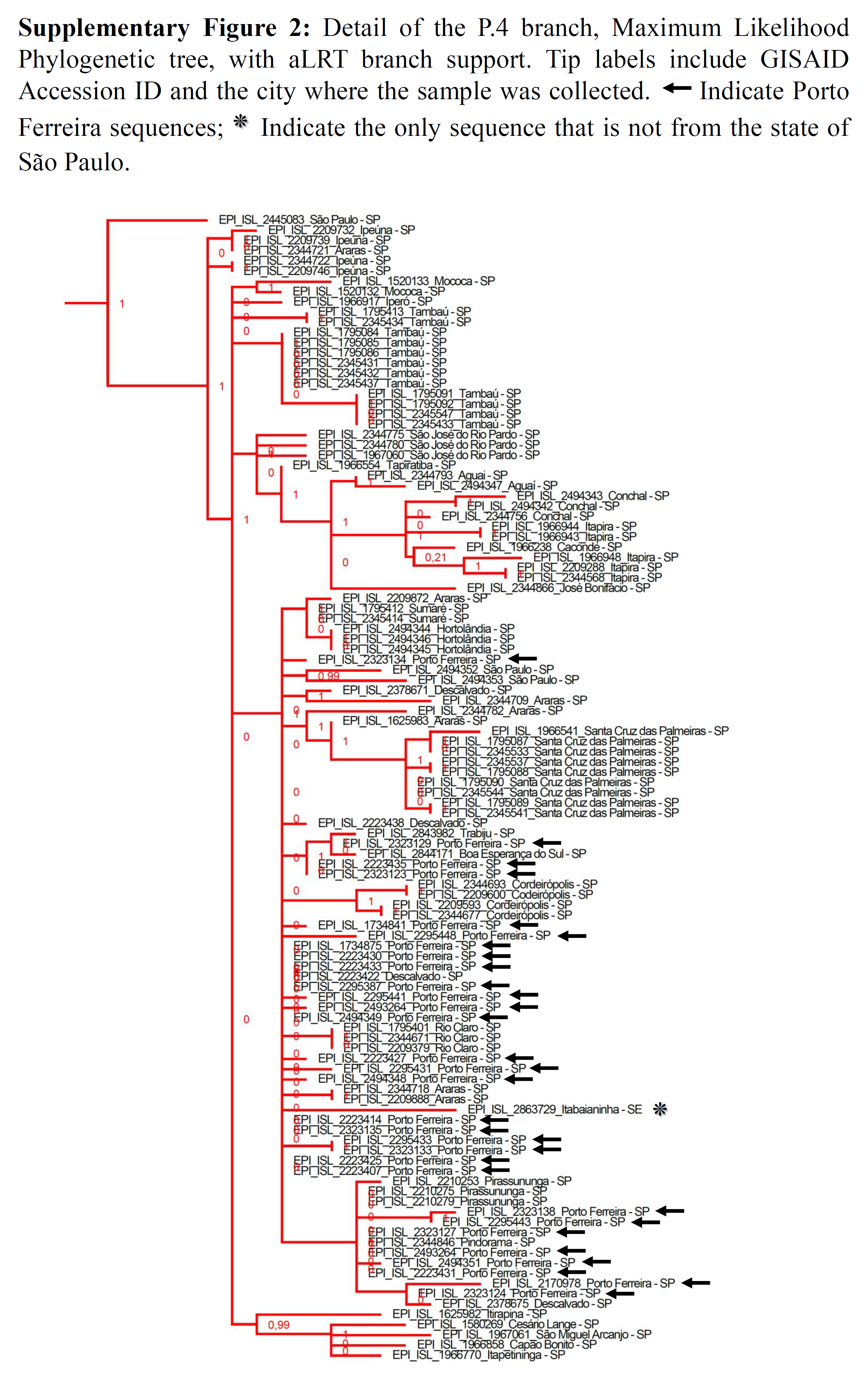

Supplement: Supplementary file 1 [file Data_Sheet_1.zip › Supplentary/Supplementary Figure 2.tif]
